# Anxiety and Depression as Risk Factors for Vasovagal Syncope and Potential Treatment Targets: A Systematic Review

**DOI:** 10.7759/cureus.32793

**Published:** 2022-12-21

**Authors:** Baraa Abuzainah, Sai Dheeraj Gutlapalli, Dipabali Chaudhuri, Kokab Irfan Khan, Roba Al Shouli, Akhil Allakky, Asila A Ferguson, Aujala Irfan Khan, Pousette Hamid

**Affiliations:** 1 General Practice, California Institute of Behavioral Neurosciences and Psychology, Fairfield, USA; 2 Internal Medicine, California Institute of Behavioral Neurosciences and Psychology, Fairfield, USA; 3 Research, California Institute of Behavioral Neurosciences and Psychology, Fairfield, USA; 4 Pediatrics, California Institute of Behavioral Neurosciences and Psychology, Fairfield, USA; 5 Infectious Disease, California Institute of Behavioral Neurosciences and Psychology, Fairfield, USA; 6 Psychiatry, California Institute of Behavioral Neurosciences and Psychology, Fairfield, USA; 7 Neurology, California Institute of Behavioral Neurosciences and Psychology, Fairfield, USA

**Keywords:** transient loss of consciousness, reflex syncope, vasovagal syncope, depression, anxiety

## Abstract

Vasovagal syncope (VVS) is a transient, sudden loss of consciousness followed by complete resolution, usually due to a paradoxical autonomic reaction that results in hypotension and/or bradycardia. In this study, we assessed the correlation between VVS and a patient’s psychiatric status, as well as if this association could be a target in the treatment of those patients. We conducted a systematic review in accordance with the Preferred Reporting Items for Systematic Reviews and Meta-Analyses (PRISMA) guidelines, we searched the available literature using the following databases: PubMed, Google Scholar, and ScienceDirect, with last access on July 21, 2022. The search resulted in 1691 articles, and inclusion and exclusion criteria were applied to nine remaining articles, all of which were accepted after using the quality assessment tools, four observational and four randomized controlled trials (RCTs). Four of the included studies assessed the correlation among vasovagal syncope, psychosocial impairment, and quality of life. We found a consistent correlation among VVS, psychosocial impairment, and quality of life (QoL), meaning that VVS patients usually have some degree of psychosocial impairment, especially in the form of anxiety and depression, and a poorer QoL in comparison to their healthy counterparts. The use of psychotherapy and antidepressants was proven to be effective in VVS in RCTs, but further evidence is needed.

## Introduction and background

Syncope is a transient and sudden loss of consciousness followed by complete retention of function [1]. It’s a very common problem with an estimated lifetime prevalence of around 40%. It’s also considered a financial burden with the median cost of hospitalization around $8679 [2]. Vasovagal syncope (VVS) is the most common cause of syncope [3], with around one-third of all people having at least one syncopal episode during their lifetime [4]. The exact mechanism of VVS has not been identified, with the main hypothesis suggesting a paradoxical reaction of the autonomic nervous system that causes hypotension and/or bradycardia [5]. This mechanism that is responsible for VVS differs from baroreceptor-driven blood pressure control by at least two cardinal aspects: first, VVS usually results in hypotension, but the trigger that sets it in motion is not hypertension like is the case in baroreceptor reflex, and second is that blood pressure and heart rate decrease together in most forms of VVS and this reversal of the usual reciprocal behavior indicates that normal baroreceptor reflex is lost during the syncopal episode of VVS [6].

The diagnosis of VVS is mainly dependent on history and physical examination. Tilt-table testing has been identified as a safe and reliable modality to show susceptibility to VVS, especially in cases where history alone is undiagnostic [7]. Considering the benign nature of VVS, treatment is rarely needed, but in some patients, conservative measures alone are unsatisfactory and effective treatment is needed, as syncopal episodes may result in injury and they may affect the quality of life [8]. Despite the need and efforts of scientists, there are limited therapeutic options for this condition. The treatment of VVS depends primarily on patient education about the condition and its prognosis, non-pharmacological therapies can be used to prevent the symptoms and to prevent syncope at the onset of presyncope, and, if needed, pharmacological options are also available, with many of which focused on blood volume expansion and vasopressor effect [9]. Cardiac pacing is also another treatment option in certain patients.

The association between different psychiatric conditions, especially anxiety and depression, and VVS and whether anxiety and depression are risk factors for the development of VVS or they are just an outcome, it’s still not fully understood. Establishing this relationship will help physicians develop a clearer understanding of this condition, and will allow them to investigate other treatment options. In this study, we have conducted a systematic review of the best available evidence related to this topic, and we hope to establish clearer and more consistent evidence regarding the association between VVS and psychiatric illness as well as the benefit of using some psychiatric medications in the treatment of VVS.

## Review

Methodology

This systematic review was carried out in the accordance with the Preferred Reporting Items for Systematic Reviews and Meta-Analyses (PRISMA) guidelines [10]. Search strategy along with inclusion and exclusion criteria are discussed below.

*Databases* 

Systemized search of the articles was done in PubMed, Google Scholar, and ScienceDirect. Only articles published in the English language were considered. The last search was done on July 21, 2022. The following search terms were used in combination: “anxiety,” “depression,” and “vasovagal syncope.” On PubMed, we used the following medical subject headings (MeSH) keywords: generalized anxiety disorder OR generalized anxiety disorder (GAD) OR anxiety OR stress AND Major depressive disorder OR depression OR major depressive disorder (MDD) OR sadness AND vasovagal syncope OR reflex syncope OR syncope OR loss of consciousness OR transient loss of consciousness AND anxiety ({“anxiety/complications” (Majr) OR “anxiety/drug effects” (Majr) OR “anxiety/drug therapy” (Majr) OR “anxiety/history” (Majr) OR “anxiety/pathology” (Majr) OR “anxiety/therapy” (Majr)}) OR (“anxiety/complications” {Mesh:NoExp} OR “anxiety/drug effects” {Mesh:NoExp} OR “anxiety/drug therapy” {Mesh:NoExp} OR “anxiety/history” {Mesh:NoExp} OR “anxiety/pathology”{Mesh:NoExp} OR “anxiety/therapy” {Mesh:NoExp}) AND depression ({“depression/complications” (Majr) OR “depression/diagnosis” (Majr) OR “depression/etiology” (Majr) OR “depression/history” (Majr) OR “depression/pathology” (Majr) OR “depression/prevention and control” (Majr) OR “depression/psychology” (Majr) OR “depression/rehabilitation” (Majr) OR “depression/therapy” (Majr)}) OR (“depression/complications”{Mesh:NoExp} OR “depression/diagnosis” {Mesh:NoExp} OR “depression/etiology” {Mesh:NoExp} OR “depression/history” {Mesh:NoExp} OR “depression/pathology” {Mesh:NoExp} OR “depression/prevention and control” {Mesh:NoExp} OR “depression/psychology” {Mesh:NoExp} OR “depression/rehabilitation” {Mesh:NoExp} OR “depression/therapy” {Mesh:NoExp}) AND vasovagal syncope ({“syncope, vasovagal/complications” (Majr) OR “syncope, vasovagal/diagnosis” (Majr) OR “syncope, vasovagal/drug therapy” (Majr) OR “syncope, vasovagal/etiology” (Majr) OR “syncope, vasovagal/history” (Majr) OR “syncope, vasovagal/pathology” (Majr) OR “syncope, vasovagal/prevention and control” (Majr) OR “syncope, vasovagal/psychology” (Majr) OR “syncope, vasovagal/rehabilitation” (Majr) OR “syncope, vasovagal/therapy” (Majr)}) OR (“syncope, vasovagal/complications” {Mesh:NoExp} OR “syncope, vasovagal/diagnosis” {Mesh:NoExp} OR “syncope, vasovagal/drug therapy” {Mesh:NoExp} OR “syncope, vasovagal/etiology” {Mesh:NoExp} OR “syncope, vasovagal/history” {Mesh:NoExp} OR “syncope, vasovagal/pathology” {Mesh:NoExp} OR “syncope, vasovagal/prevention and control” {Mesh:NoExp} OR “syncope, vasovagal/psychology” {Mesh:NoExp} OR “syncope, vasovagal/rehabilitation” {Mesh:NoExp} OR “syncope, vasovagal/therapy” {Mesh:NoExp}).

Eligibility Criteria and Study Selection 

Two independent investigators (BA, SG) independently screened each article by abstract and title for eligibility, then articles included by each investigator were compared and any disagreement was resolved by discussion, if no consensus can be reached, a third independent investigator (DC) decided the eligibility.

Inclusion criteria and exclusion criteria were as follows: the studies included were (1) free full texts, (2) study type - cohort, cross-sectional, case-control, and randomized controlled trial (RCT), (3) studies conducted on humans, (4) studies in the English language, and (5) studies conducted on adults. Only studies meeting these criteria were included in the final review. The studies excluded were (1) paid articles or abstracts, (2) books and documents, case reports, and review articles, and (3) studies conducted on pediatric patients.

Data Extraction

Two researchers (BA, SG) independently extracted data from the included studies using a standardized form. The form included are as follows: study design, number of participants, mean age, gender percentage, median number of total syncopal episodes (TSE), type of syncope in included patients, and aim of the study.

Quality Assessment Tools 

Two independent investigators (BA, SG) screened each one of the remaining articles for risk of bias using commonly available quality assessment tools. Newcastle-Ottawa quality assessment scale for case-control and cohort studies, we included all studies with a score of six or more (Table 1). The revised Cochrane risk-of-bias tool for randomized trials (RoB 2) was used for randomized controlled trials (RCTs) (Table 2). Studies with low risk or some concerns of bias were accepted, and studies with high risk of bias were rejected. Any disagreement was resolved by consensus.

**Table 1:** Quality assessment of observational studies using the Newcastle-Ottawa questionnaire. *Asterisk represents the score of the assessment tool. Each asterisk indicates one point.

Study	Selection	Comparability	Outcome/exposure	Overall (max 9)
Giada et al. (2005) [11]	****	**	***	9, good
Ng et al. (2019) [12]	****	*	***	7, good
Atici et al. (2020) [13]	****	-	***	6, good
Flint et al. (2009) [14]	****	**	***	9, good
Kaya et al. (2007) [15]	***	-	***	6, good

**Table 2:** Quality assessment of RCT using the Cochrane risk-of-bias tool. RCT: randomized controlled trial; D1: domain 1 (risk of bias arising from the randomization process); D2: domain 2 (risk of bias due to deviations from the intended interventions); D3: domain 3 (missing outcome data); D4: domain 4 (risk of bias in measurement of the outcome); D5: domain 5 (risk of bias in selection of the reported result)

RCT	D1	D2	D3	D4	D5	Overall
Di Girolamo et al. (1999) [16]	Some concerns	Low risk	Low risk	Low risk	Low risk	Some concerns
Theodorakis et al. (2006) [17]	Low risk	Some concerns	Low risk	Low risk	Low risk	Some concerns
De Barros et al. (2022) [18]	Low risk	Some concerns	Low risk	Low risk	Low risk	Some concerns
Flevari et al. (2017) [19]	Some concerns	Low risk	Low risk	Low risk	Low risk	Some concerns

Results

Literature Search Strategy

**Figure 1:**
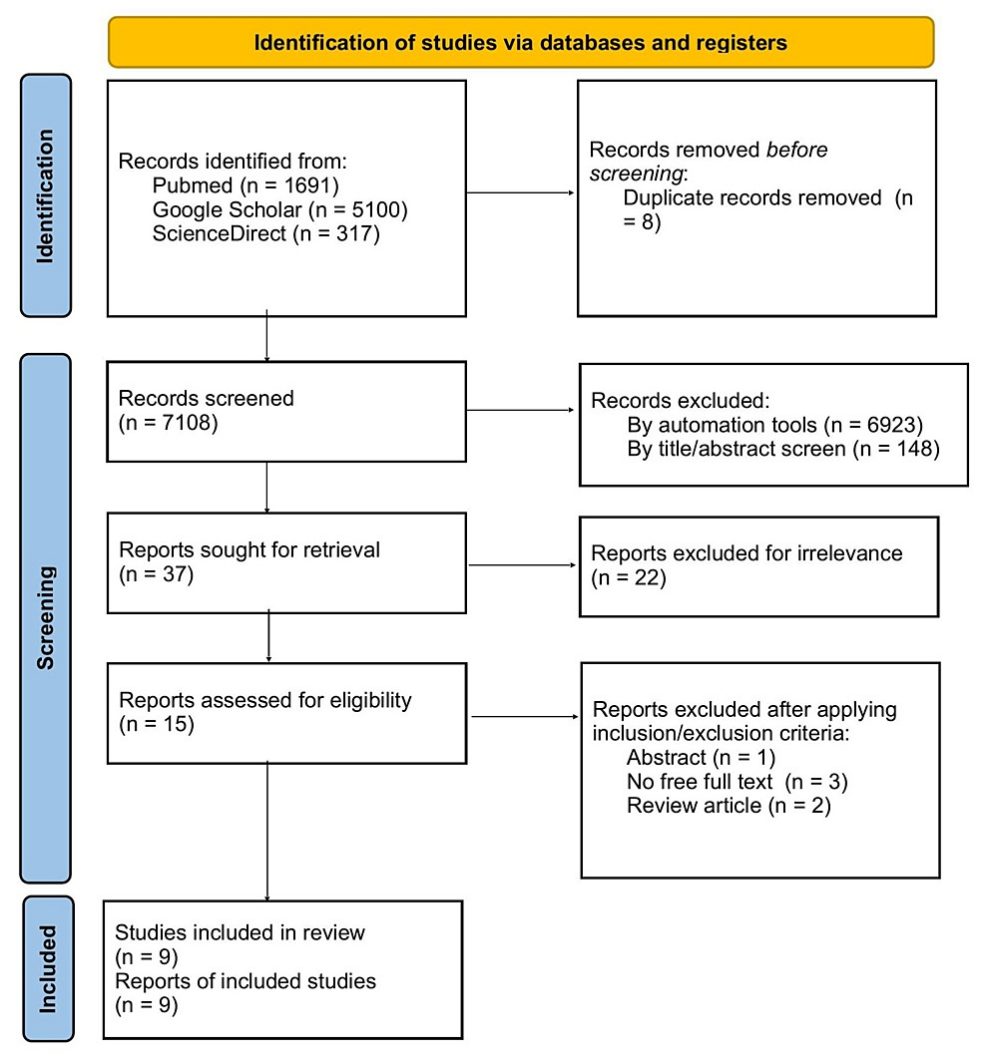
Preferred Reporting Items for Systematic Reviews and Meta-Analyses (PRISMA) flow diagram.

We used PubMed as the major electronic database for our search; we accessed PubMed on July 21, 2022, and the search resulted in 1691 articles. We also used Google Scholar which yielded 5100 results, only 90 of the first sorted by relevance were included, and ScienceDirect yielded 317 articles. Those results were filtered out using automated filters in each database and were narrowed down using inclusion and exclusion criteria to 185 articles after duplicate removal. The remaining 185 articles were screened independently by two investigators by title and abstract for eligibility and relevance, resulting in nine remaining articles (Figure 1).

Baseline Characteristics of Included Studies

We have included nine articles in this review. The details of the included articles are shown in a table (Table 3).

**Table 3:** Baseline characteristics of included studies. Types of syncope based on the modified Vasovagal Syncope International Study (VASIS) are type I (mixed), type II A (cardioinhibition without asystole), type II B (cardioinhibition with asystole), and type III (vasodepressive). TSE: total syncopal episodes, N/A: not available, VVS: vasovagal syncope, QoL: quality of life, RCT: randomized controlled trial

Study	Study design	Total number of patients	Number of patients in syncope/intervention group	Number of patients in control/ placebo group	Mean age of patients in syncope/intervention group	Mean age of patients in control/placebo group	Percentage of female patients in syncope/ intervention group	Percentage of female patients in control/placebo group	Median number of TSE	Syncope type	Mean follow-up period	Aims of the study
Giada et al. (2005) [11]	Case-control	122	61	61	44	41	67%	67%	7	N/A	N/A	Assesses the correlation between VVS, QoL, and psychological distress
Ng et al. (2019) [12]	Cross-sectional	161	76	85	34	35	68%	80%	16	N/A	N/A	Assesses the correlation between VVS, QoL, and psychological distress
Atici et al. (2020) [13]	Cross-sectional	88	88	N/A	34.7	N/A	61%	N/A	10.1	Type I (49%)	N/A	Assesses the correlation between VVS, QoL, and psychological distress
										Type II (17%)		
										Type III (34%)		
Flint et al. (2009) [14]	Prospective cohort	108	108	N/A	52.3	N/A	70.4%	N/A	N/A	N/A	3 months	Assesses the correlation between psychosocial impairment and response to treatment
de Barros et al. (2022) [18]	RCT	10	5	5	48.8	46	80%	60%	N/A	Type I (80%)	1 year	Assesses the effect of psychotherapy on VVS
										Type II (20%)		
										Type III (0%)		
Di Girolamo et al. (1999) [16]	RCT	68	34	34	43.2	46.1	45%	30%	7.2/year	N/A	25.4 months	Assesses the effect of paroxetine on VVS
Theodorakis et al. (2006) [17]	RCT	76	30	22	39.3	42.8	43%	63%	5.8	N/A	6 months	Assesses the effect of fluoxetine on VVS
Kaya et al. (2007) [15]	Nonrandomized clinical trial	74	74	N/A	24	N/A	65%	N/A	3	Type I+III (91%)	6 months	Assesses the effect of amitriptyline on VVS
										Type II (9%)		
Flevari et al. (2017) [19]	RCT	106	40	20	39	41	40%	45%	6	N/A	1 year	Assesses the effect of fluoxetine on VVS

Discussion

The correlation between psychological distress and VVS has been a topic of increasing interest, especially in light of the limited efficacy of traditional treatments and the need for different approaches. In this review, we collected the best evidence available regarding this correlation and its treatment targets.

The Relationship Between VVS and Quality of Life

The correlation between VVS, QoL, and psychological distress was shown in three of the included studies. All of them agreed on a negative correlation between VVS and QoL, which indicates that patients with VVS usually have a worse QoL than their normal counterparts. Among those three studies are two cross-sectional studies, and one case-control study, so none of them was able to establish causality, meaning that VVS causes a bad QoL or that people with bad QoL are at higher risk of developing VVS [11-13].

Giada et al. conducted a case-control study on 122 participants (61 VVS patients, 61 normal controls), where they assessed the correlation between VVS and QoL using the short-form survey (SF-36 scale), this scale includes two domains - a physical domain (which includes physical functioning, physical role, bodily pain, and general health) and a psycho-social domain (which includes vitality, social functioning, emotional role, and mental health) [11]. In this study, patients with VVS had a marked reduction in all QoL scales in comparison to their healthy counterparts.

Ng et al. also agreed with the findings of this study [12]. VVS patients reported poorer quality of life compared to healthy individuals in all eight health dimensions measured by RAND36 health survey and global health visual analog scale (VAS), which includes physical functioning, general health, role limitations (physical health/emotional problems), pain, emotional well-being, social functioning, energy/fatigue, physical health composite, and mental health composite. This study, in addition to the study by Giada et al., is the only available study that used a healthy control group for comparison, but this study was superior to the study by Giada et al. by using a more diverse and representative control group of healthy volunteers, in comparison with the study by Giada et al. that used nurses in the cardiovascular department as a control group. This study was limited by using self-reported measures for QoL and psychological distress.

Atici et al. also agreed with the findings of this study [13]. In their study, they included 88 patients with VVS and assessed their QoL using EQ-5D QoL scale developed by EuroQoL group, which assesses QoL in the following five dimensions: mobility, usual activities, self-care, anxiety/depression, and pain/discomfort. The study found a significant and negative correlation between total syncopal episodes (TSE) and QoL. This study used a quantitative variable (TSE) to assess the correlation with QoL, so the linearity of the correlation could be assessed, Pearson correlation coefficient was calculated and showed a strong negative correlation of -0.649. This study was limited by the absence of a control group, and the data were self-reported and not evaluated by psychiatrists.

The Relationship Between VVS and Psychological Distress

The correlation between VVS and psychological distress was shown in three studies, and all of them emphasized that patients with VVS have more psychological distress in comparison to healthy controls. The study by Giada et al. showed a higher incidence of psychological distress, especially anxiety, depression and other mood disorders, somatization, and hysteria among VVS patients in comparison with healthy individuals [11]. They also found that the presence of psychiatric disorder was a risk factor for syncopal recurrence, but they found no correlation between the patient’s syncopal burden and psychiatric diagnosis. Those findings were also emphasized by the study by Ng et al., which showed a higher incidence of anxiety and depression among patients with VVS, but this study has a larger sample size and used a different assessment tool [12]. In the study by Ng et al., anxiety sensitivity, which is linked to the perception that anxiety is linked to negative effects, has shown to be greater in patients with VVS.

The study by Atici et al. also had similar findings, by investigating the correlation between VVS and anxiety and depression using Beck depression and Beck anxiety scales [13]. They also found that Beck-anxiety and Beck-depression scales were independent parameters of total syncopal episodes (TSE).

The study by Flint et al. found a correlation between psychosocial impairment and response to treatment [14], which supports the recommendation that patients with VVS, who presents with psychosocial impairment should undergo a psychiatric evaluation by a therapist, especially in the light of other evidence that indicated the usefulness of psychotherapy and antidepressants in the treatment of refractory VVS [15-19]. This study was limited by the use of self-report measures for the psychosocial evaluation, and it didn’t control for other co-morbidities that can affect the psychiatric condition of the patients.

The Effect of Psychotherapy

There was a gap in the literature regarding the effect of psychotherapy on VVS patients, the only study we found was a recent RCT conducted by de Barros E Silva et al. on a small number of patients (five in the intervention group, five in the control group) [18]. In this trial, they evaluated the effect of once-a-week psychotherapy sessions on five patients with VVS, who are already on the conventional treatment of VVS as indicated in the international guidelines but they still have recurrent episodes of syncope refractory to their treatment. In this study, they found a statistically significant effect of psychotherapy on the recurrence of syncopal and pre-syncopal episodes over one year and a positive effect on QoL. Although this study was limited by the small number of patients which will affect its external validation, it can be considered as a proof of concept and indicates that psychotherapy should be considered, especially in patients refractory to the conventional treatment. interestingly, during the follow-up period of patients, they found that all the patients in the intervention group have a history of trauma associated with their first syncopal episodes, which also will limit the external validation of this study.

The Use of Antidepressants

The association between psychological distress and VVS may indicate a common pathophysiological pathway, there are multiple neurotransmitters have been shown to be associated with VVS, but serotonin was among the most studied ones [20]. Some serotonin receptors in the central nervous system (CNS) have shown to be associated with blood pressure control, which indicates a clear role of serotonin in blood pressure changes observed in patients with syncope during tilt-table testing [21]. The effect of selective serotonin receptor inhibitors (SSRIs) on VVS can be explained by the down-regulation of serotonin receptors as a result of high serotonin levels induced by those medications which will result in less sensitivity to rapid fluctuations in serotonin concentrations in the central nervous system [22]. In this review, we reviewed three RCTs on the efficacy of SSRIs in the treatment of VVS, two trials used fluoxetine and one trial used paroxetine.

SSRIs have shown to be effective in the treatment of VVS patients, but this effect is seen in patients refractory to traditional therapies and with psychological distress. In the study by Girolamo et al., RCT was performed on 68 patients to assess the effect of paroxetine on refractory VVS patients [16]. The trial found a significant effect of paroxetine in preventing tilt-induced syncopal episodes as well as spontaneous syncope in patients with refractory syncope unresponsive to traditional therapies, but those findings are limited by the low reproducibility of tilt tests (positive test reproducibility 54.5%) [23].

Theodorakis et al. and Flevari et al. studied the effect of fluoxetine on VVS patients [17,19]. Theodorakis et al. trial studied the effect of fluoxetine on VVS patients referred to an outpatient clinic and was not limited to refractory cases or those with some degree of psychological distress, in this study they found no significant effect of fluoxetine therapy on the distribution of event-free time, number of syncopal episodes, pre-syncopal episodes and total events in the intention-to-treat analysis. During the follow-up period of the study, only 76 out of 96 patients continued their treatment, so the on-treatment analysis was also performed and only small significant effects were observed regarding fluoxetine use, including a decrease in the total number of patients who developed syncopal or pre-syncopal episode during their follow-up, and a decrease in the recurrence of pre-syncopal attacks. In addition, they found an improvement in well-being scores among patients in the intervention group in comparison to the control group.

The study by Flevari et al. was more selective by including only patients with refractory VVS with a positive anxiety sensitivity index (ASI) [19]. They found that fluoxetine therapy in VVS patients with recurrent syncopal episodes and anxiety sensitivity was more effective than placebo and can be used as a first-line treatment. The patients receiving fluoxetine therapy had a statistically significant difference in the distribution of syncope-free periods. This finding indicates that the effect of SSRIs is not universal to all patients and it should be individualized to a specific group of patients. This study was limited by the absence of a standardized education protocol in addition to the standard of care for all the included patients.

The use of other antidepressants in the treatment of VVS was less commonly investigated. The study by Kaya et al. assessed the effect of amitriptyline, a tricyclic antidepressant, on 74 patients with VVS [15]. The study found a positive effect of amitriptyline in the prevention of syncopal and pre-syncopal episodes, as assessed by a repeat tilt-table test after one month and the occurrence of any spontaneous episodes during a six-month follow-up period. This study has no control group, so randomized controlled trials are still needed to assess those findings, as well as, the utility of a repeat tilt-table test in the assessment of syncope recurrence is limited by its low reproducibility.

Limitations

This study was limited by a number of factors. First is the limited number of available relevant articles, with the possibility of missing some related articles; due to our research strategy. Second, the absence of uniform assessment tools for QoL and psychological status throughout the included studies.

## Conclusions

In conclusion, patients with vasovagal syncope have a higher incidence of psychiatric distress than normal people, especially in the form of anxiety and depression. They also have poorer quality of life when compared to healthy controls. However, with the available evidence, no causality can be established. Moreover, patients with VVS were found to benefit from the use of psychotherapy and antidepressants, especially paroxetine and fluoxetine, as part of their treatment regimen, but this benefit was mostly noticed in patients with some degree of psychosocial impairment. Those findings should draw more attention to the psychological status in the treatment of VVS. We support the recommendation that all patients with VVS should undergo a full psychological assessment by an experienced psychiatrist or primary care physician, and patients with psychosocial impairment should be considered for the use of psychotherapy and/or antidepressants. Further high-quality, large-sample observational and RCT are needed to strengthen this available evidence.
